# Construction and analysis of a joint diagnosis model of random forest and artificial neural network for heart failure

**DOI:** 10.18632/aging.202405

**Published:** 2020-12-26

**Authors:** Yuqing Tian, Jiefu Yang, Ming Lan, Tong Zou

**Affiliations:** 1 Peking University Fifth School of Clinical Medicine, Beijing 100730, P.R. China; 2 Department of Cardiology, Beijing Hospital, National Center of Gerontology, Institute of Geriatric Medicine, Chinese Academy of Medical Science, Beijing 100730, P.R. China

**Keywords:** difference analysis, heart failure, artificial neural network, random forest

## Abstract

Heart failure is a global health problem that affects approximately 26 million people worldwide. As conventional diagnostic techniques for heart failure have been in practice with various limitations, it is necessary to develop novel diagnostic models to supplement existing methods. With advances and improvements in gene sequencing technology in recent years, more heart failure-related genes have been identified. Using existing gene expression data in the Gene Expression Omnibus (GEO) database, we screened differentially expressed genes (DEGs) of heart failure and identified six key genes (*HMOX2*, *SERPINA3*, *LCN6*, *CSDC2*, *FREM1*, and *ZMAT1*) by random forest classifier. Of these genes, *CSDC2*, *FREM1*, and *ZMAT1* have never been associated with heart failure. We also successfully constructed a new diagnostic model of heart failure using an artificial neural network and verified its diagnostic efficacy in public datasets.

## INTRODUCTION

Heart failure (HF) is a chronic condition common to all types of heart disease [1]. In HF, which is essentially a pathophysiological state caused by abnormal heart functions, the heart cannot meet the pumping speed required for normal metabolism under normal heart pressure [2]. HF is categorized into two types of diseases: one is HF with reduced ejection fraction (HFrEF) and the other is HF with preserved ejection fraction (HFpEF). HF with mid-range ejection fraction is more contentious and not included in our current study. The mechanisms that are involved in the occurrence and development of these two types of HF are obviously different.

HFrEF is mostly caused by initial myocardial damage and disease conditions that affect ventricular contraction. These disease conditions may originate from cardiovascular diseases themselves or may be secondary cardiovascular dysfunction caused by diseases related to other organ systems [3]. Approximately two-thirds of HFrEF cases are caused by coronary artery disease [3]. The occurrence and developmental process of HFrEF are complex and includes the following changes, as revealed by microscopic analyses: 1) changes in the structure of cardiomyocytes, such as glycogen deposition and sarcomere depletion; 2) abnormal sodium and potassium channels in cardiomyocytes; 3) abnormal energy metabolism in cardiomyocytes, such as increased glucose utilization and decreased oxidative phosphorylation; and 4) other mechanisms, including oxidative stress, apoptosis, and autophagy [4]. From a pathophysiological perspective, initial myocardial damage causes stress reactions in undamaged myocardium, such as myocardial cell apoptosis, hypertrophy, and collagen fibril deposition, which lead to hypofunction of the cardiac pump, reduced cardiac output, and decreased blood perfusion in tissues and organs, and eventually cannot meet the metabolic needs of the body [3]. These pathophysiological processes result in activation of neurohumoral regulation mechanisms to maintain the pumping function of the heart, mainly via the sympathetic nervous system and the renin–angiotensin–aldosterone system. However, long-term activation of the neurohumoral regulatory mechanism stimulates remodeling of the ventricles, endothelin secretion, and cytokine upregulation, which in turn causes vasoconstriction and cardiac overload, and results in a vicious circle [3].

HFpEF often occurs in pressure-overload hypertrophy diseases [5]. Compared with HFrEF, HFpEF is more likely to decrease in cardiac reserves [6]. Considering pathophysiological mechanisms, left ventricular diastolic dysfunction, especially increased left ventricular filling pressure (LVFP), is the most common early manifestation among these patients. In the early stage of the disease, increased LVFP may occur only during exercise. However, the increase in LVFP becomes persistent in the progression of HFpEF [6]. Persistent diastolic dysfunction of the left ventricle may impair left atrial function and cause pulmonary hypertension, which further leads to right heart insufficiency and eventually manifests as dysfunction of the systemic circulatory system [7]. In terms of the pathogenic mechanisms involved in the development of HFpEF, cardiomyocytes themselves undergo apoptosis to a lesser extent, whereas the characteristic changes are the proliferation of abnormal fibroblasts and the accumulation of cell matrix proteins [5]. This is the most prominent difference between HFpEF and HFrEF.

**Figure 1:**
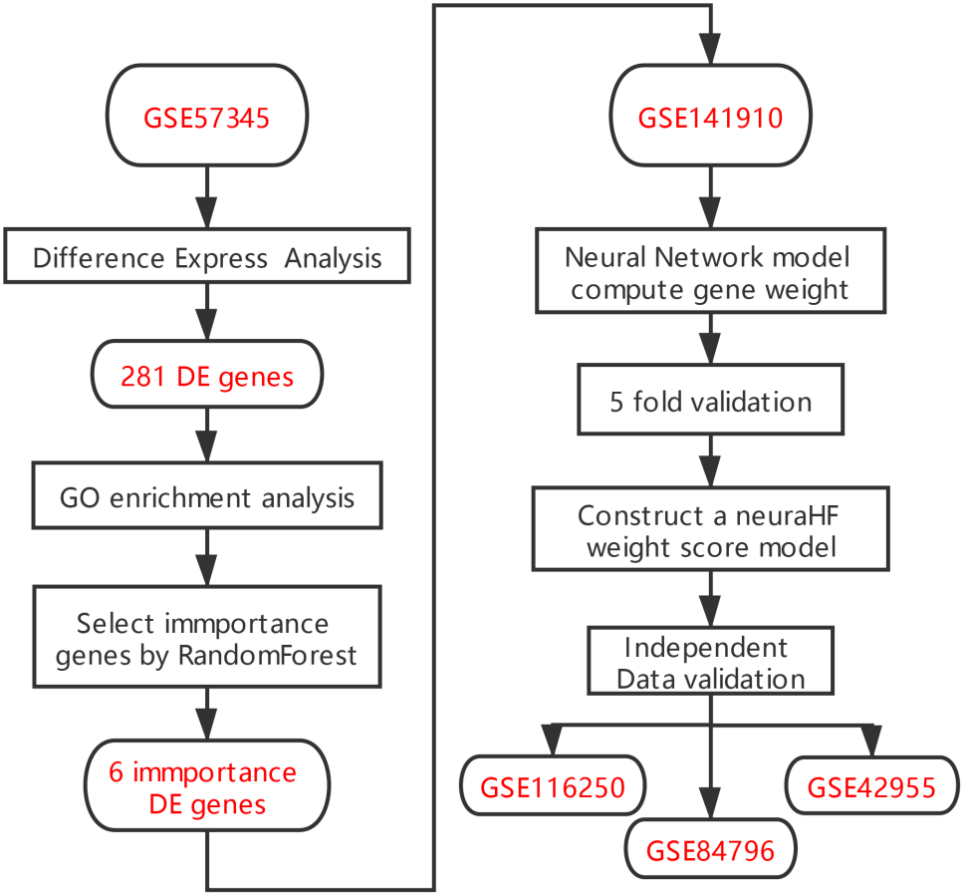
**Flowchart.**

There are several limitations associated with the diagnostic techniques for HF commonly used in clinics. The levels of brain natriuretic peptide/N-terminal-proB-type natriuretic peptide may also be elevated in various non-HF diseases, such as pulmonary hypertension, cirrhotic ascites, acute or chronic renal failure, infection, and inflammation [8], but normal in patients with HFpEF [7]. Echocardiography, which is another commonly used technique for the evaluation of cardiac function, relies more on the individual operation proficiency and diagnostic experience of specialists, making the examination poorly reproducible. Moreover, it is difficult to identify patients with HFpEF by simply measuring the EF value [7]. Therefore, it is necessary to develop new diagnostic models to supplement these current methods. The rapid development of second generation sequencing in recent years facilitates the identification of marker genes associated with a variety of diseases, providing a solid basis for establishing new gene-related diagnostic models of HF. In this study, we screened differentially expressed genes (DEGs) between HF and normal myocardium samples in the Gene Expression Omnibus (GEO) database. On the basis of these DEG data, we used the random forest algorithm to identify the key genes expressed in HF. We then input these key genes in artificial neural networks to construct a genetic diagnostic model of HF (See analysis process in Figure 1).

## RESULTS

### Differential expression analysis

**Figure 2:**
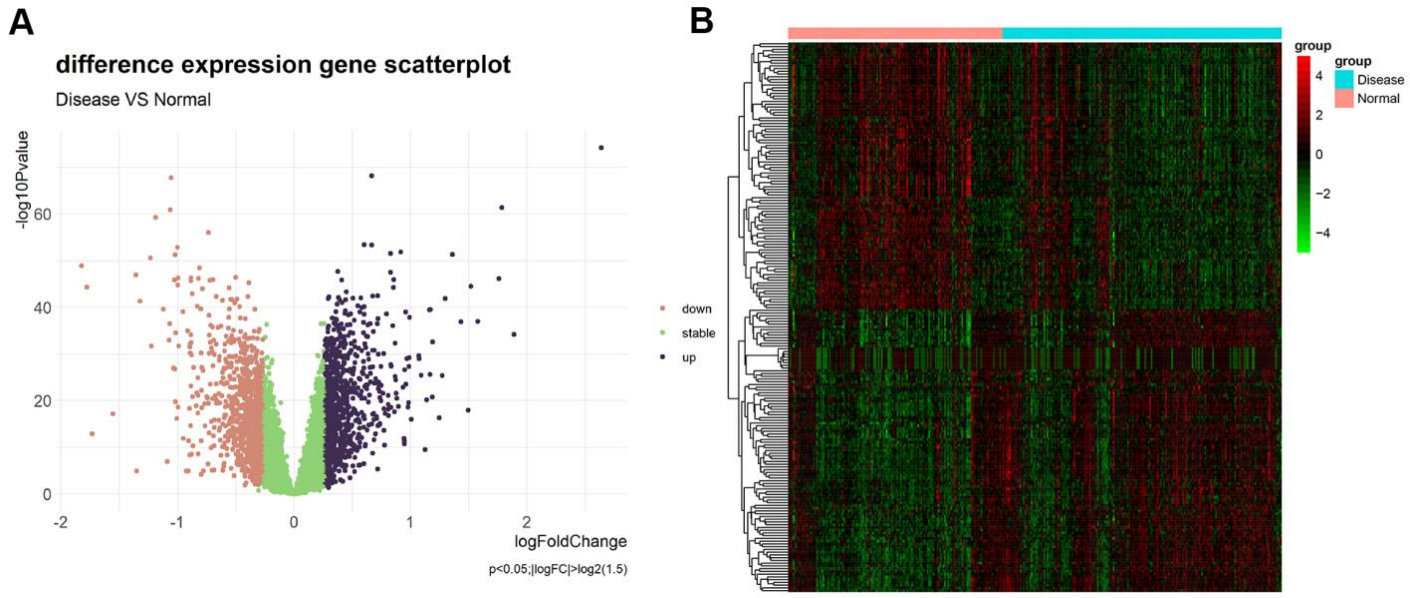
(**A**) Volcano plot of differential expression analysis results. The abscissa is logFC and the ordinate is –log10 *P* value. The upper right part has a *P* value less than 0.01 and a fold change greater than 1.5, indicating significant DEGs with higher expression levels. The upper left part has a *P* value less than 0.01 and a fold change less than −1.5, indicating significant DEGs with reduced expression. The green dots represent the remaining stable genes. (**B**) Heatmap of DEGs. The colors in the graph from red to green indicate high to low expression. On the upper part of the heatmap, the red band indicates the disease samples and the blue band indicates the normal samples.

Differential expression analysis was performed based on the chip dataset GSE57345 to screen for DEGs. The GSE57345 dataset contained 313 samples, including 136 normal and 177 HF disease samples. Next, the limma package was used to identify DEGs between the HF samples of this chip dataset and the normal control samples through the Bayesian test. The results of the DEGs are shown in the volcano graph (Figure 2A) and heatmap (Figure 2B). Based on fold change values of >1.5 and significance threshold of *P* <0.05, we identified 281 significant DEGs related to HF diseases by the screen (Supplementary File 1).

### GO/Kyoto encyclopedia of genes and genomes (KEGG) enrichment analysis

**Figure 3:**
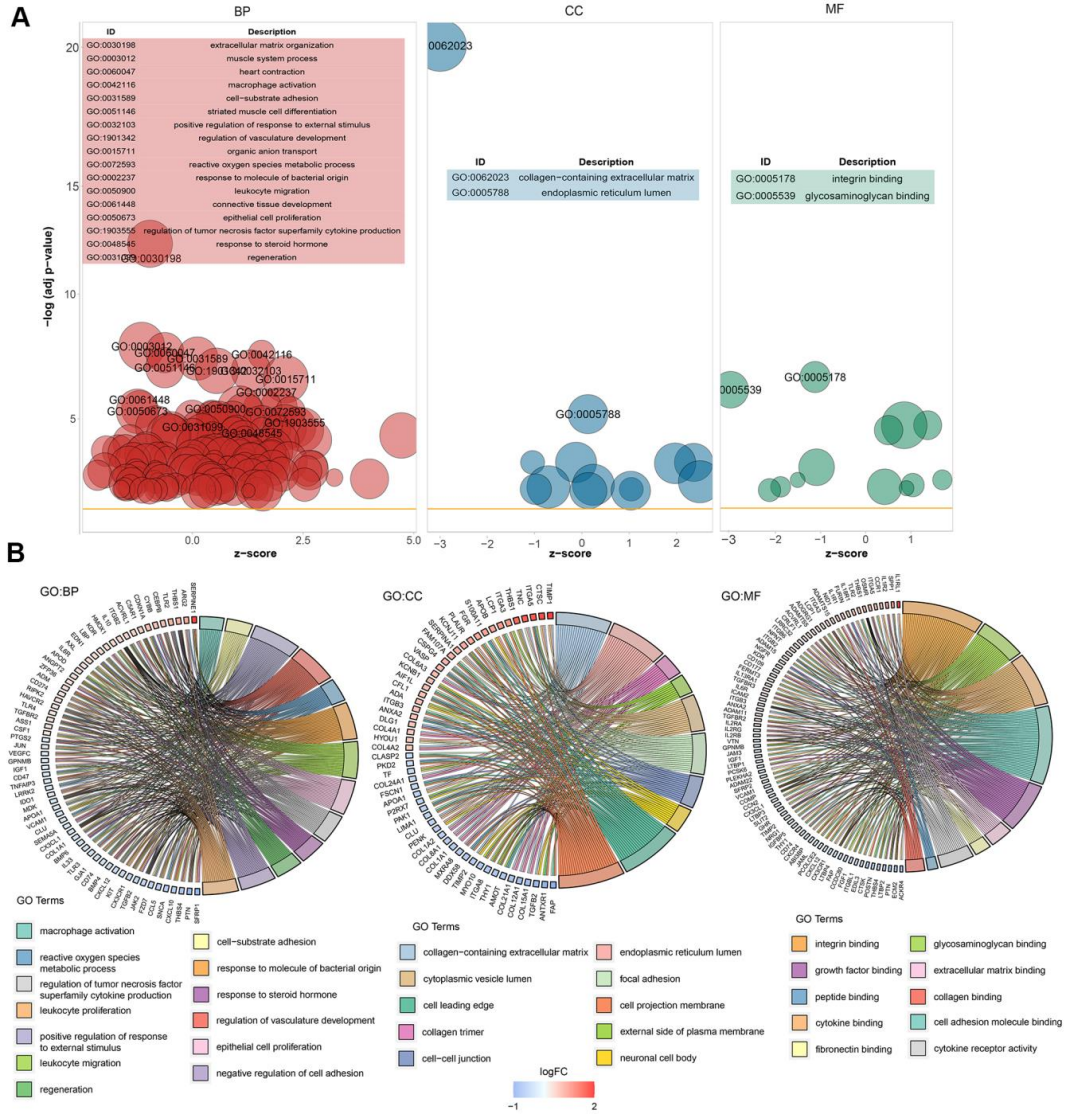
**Graph showing the enrichment analysis results.** (**A**) Bubble plot of GO enrichment results. Biological processes are shown on the left, cellular components are shown in the middle, and molecular function is shown on the right. The *x*-axis represents the *z*-score, and the *y*-axis indicates the −log10(adj *P*) values. A bubble represents a GO term, with the size of the bubble indicating the number of genes in the GO term. The results after deduplication of the GO enrichment results are shown, and the threshold is 75% coverage. The GO terms with –log10(adj *P*) > 5 are marked and shown in the table. (**B**) Ring plot showing GO enrichment. The left side indicates the DEGs, the red gene band indicates upregulation, and blue indicates downregulation. The band on the right with different colors represents different GO terms. The connecting line indicates that the gene is included in the GO term.

GO enrichment analysis was performed on the 281 significant DEGs using the clusterProfiler package. The Benjamini–Hochberg correction method was used, with the thresholds set at a *P* value of <0.01 and a Q value of <0.01. To avoid redundancy in the GO enrichment results, we performed deduplication on the GO enrichment terms and eliminated terms with a gene overlap of >0.75 (Supplementary File 2). Figure 3 shows the analysis results of three aspects of GO enrichment, including biological processes, cellular components, and molecular function. Figure 3A shows the GO enrichment results of all three classifications (only the GO term results of –log10(adj P)>5 are shown). Among the results, the related biological processes involved in HF include extracellular matrix organization, heart contraction, macrophage activation, and cell–substrate adhesion. The cellular components involved include collagen-containing extracellular matrix. The molecular functions included integral binding and other important functions. Figure 3B shows part of the GO enriched terms and the significant DEGs involved. We also performed KEGG pathway enrichment analysis on the DEGs, as shown in Supplementary Figure 1, which shows the results of significant enriched biological pathways involved and the corresponding DEGs.

### Random forest screening for DEGs

**Figure 4:**
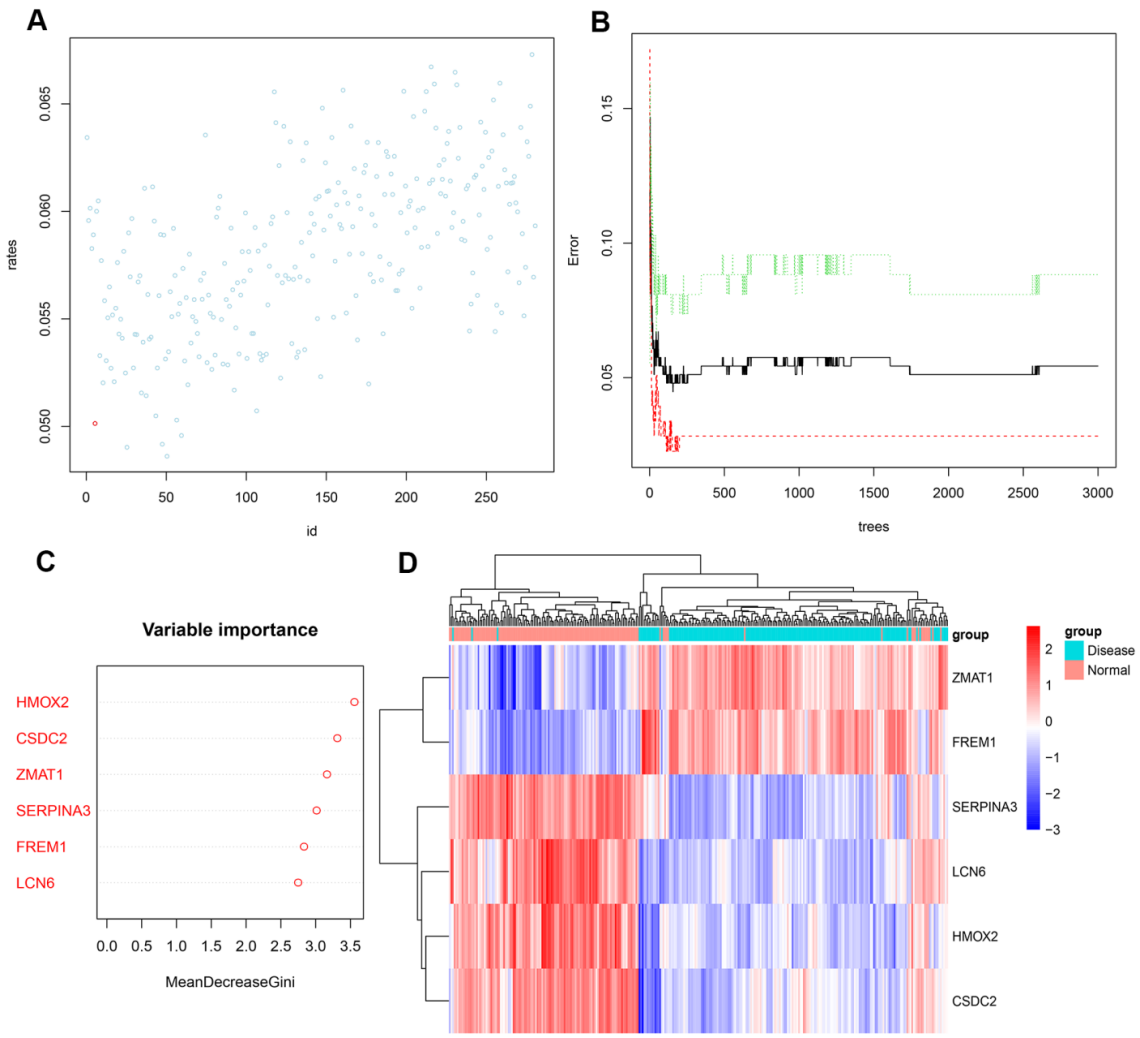
(**A**) Scatter plot of the effect of variable number selection on the average error rate. The *x-*axis represents the number of variables, and the *y*-axis indicates the out-of-band error rate. The point in the lower left represents the number of variables (i.e., six). (**B**) The influence of the number of decision trees on the error rate. The *x-*axis represents the number of decision trees, and the *y*-axis indicates the error rate. When the number of decision trees is approximately2000, the error rate is relatively stable. (**C**) Results of the Gini coefficient method in random forest classifier. The *x*-axis indicates the genetic variable, and the *y*-axis represents the importance index. (**D**) Heatmap of unsupervised clustering showing the results of the hierarchical clustering produced by the six important genes generated by random forest in GSE57345. Red color indicates genes with high expression in the samples, blue color indicates genes with low expression in the samples, the red band on the upper side of the heatmap indicates normal samples, and the blue band indicates HF disease samples.

Next, we input the 281 DEGs into the random forest classifier. To find the optimal parameter mtry (i.e., to specify the optimal number of variables for the binary trees in the nodes), we performed a recurrent random forest classification for all possible numbers among the 1–281 variables and calculated the average error rate of the model. Figure 4A shows the average error rate when all variables were selected. Finally, we chose 6 as the parameter of variable number. The number of variables was as small as possible, and the out-of-band error was as low as possible. Referring to the relationship plot between the model error and the number of decision trees (Figure 4B), we selected 2000 trees as the parameter of the final model, which showed a stable error in the model. In the process of constructing the random forest model, the variable importance of the output results (Gini coefficient method) was measured from the perspective of decreasing accuracy and decreasing mean square error (see Supplementary File 3 for the importance output results). We then identified six DEGs with an importance greater than 2 as the candidate genes for subsequent analysis. Figure 4C shows that among the six variables, *HMOX2* and *CSDC2* were the most important, followed by *ZMAT1*, *SERPINA3*, *FREM1*, and *LCN6*. Based on these six important variables, we performed k-means unsupervised clustering of the GSE57345 dataset. Figure 4D shows that the six genes could be used to distinguish between the disease and normal samples in 313 samples of the GSE57345 dataset. Among them, *ZMAT1* and *FREM1* genes are a cluster with low expression in the normal samples and high expression in the disease samples. On the other hand, *SERPINA3*, *LCN6*, *HMOX2*, and *CSDC2* belong to another cluster with high expression in the normal samples and low expression in the disease samples.

### Construction of the artificial neural network model

We used another dataset of GSE141910 to construct an artificial neural network model based on the neuralnet package. The first step was data preprocessing, which was performed to normalize the data. Next, the min-max method was selected [0,1], and was pressed to separate the zoom data before training the neural network. Before starting the calculation, the maximum and minimum data values were standardized and the number of hidden layers was set as 5. In the choice of parameters, there was no fixed rule on how many layers and neurons were to be used. The number of neurons should be between the input layer size and the output layer size, usually two-thirds of the input size. Thus, the parameter of number of neurons was set as 6. To more effectively evaluate the results of the neural network model, we selected a 5-fold cross-validation method. The dataset was randomly divided into a training set and a verification set. The purpose of the training set was to determine the weights of candidate DEGs. The verification set was used to verify the classification efficiency of the model score constructed with gene expression and gene weight. The calculation formula of the classification score of the obtained disease neural network model is as follows:

$$\begin{array}{c} \text{neuraHF}=\\ \sum \left(Gene\;Expression\times Neural\;Network\;Weight\right) \end{array}$$

**Figure 5:**
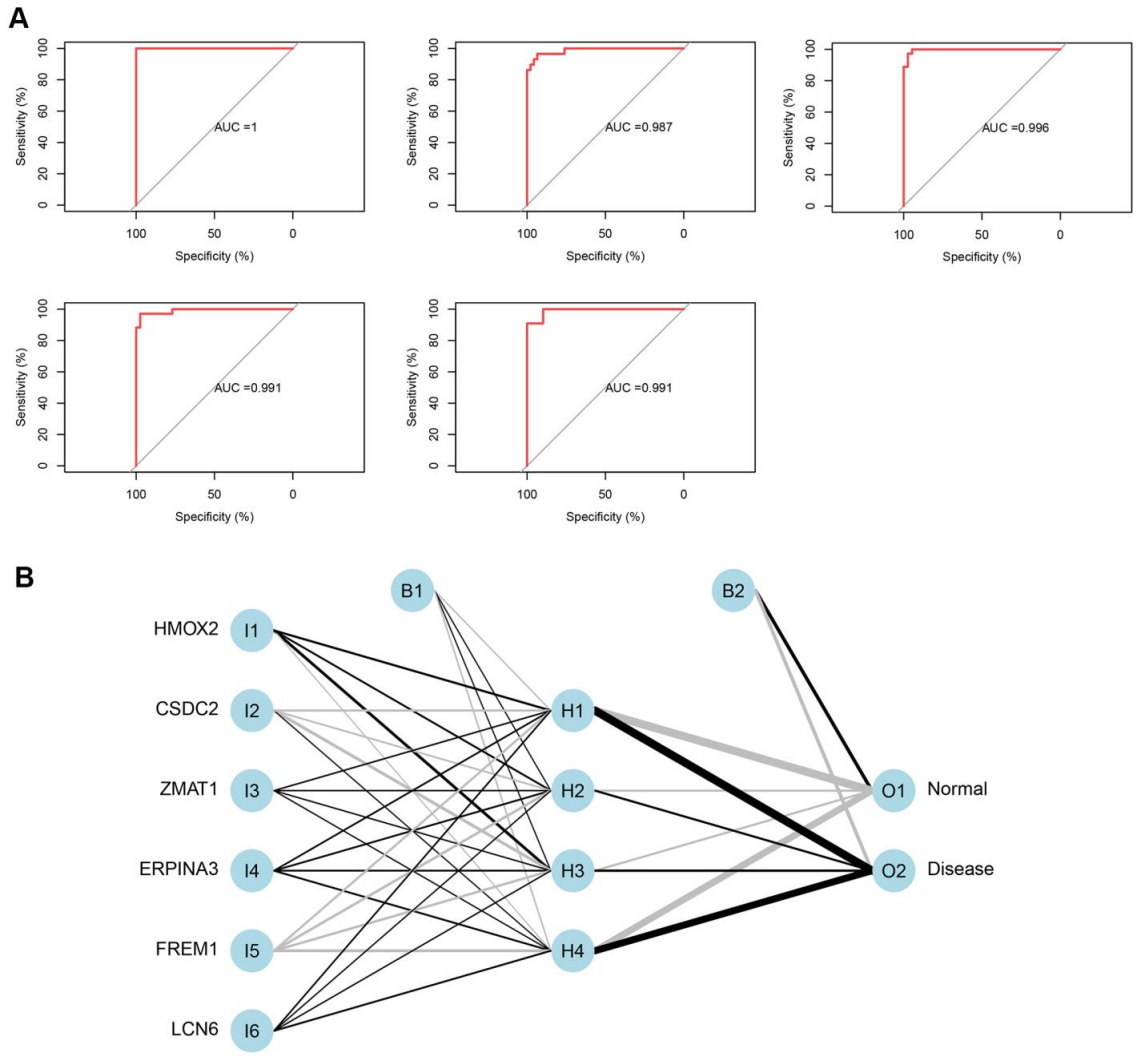
(**A**) Verification of the ROC curve results by the five-time cross-validation model. (**B**) Results of neural network visualization.

The 5-time cross-validation results display the model classification performance using the receiver operating characteristic (ROC) curve (Figure 5A). In addition, a confusion matrix was used to evaluate the predicted performance (Table 1). The areas under the ROC curves (AUC) of the five-time cross-validation results were close to 1 (average AUC > 0.99), which shows the robustness of the model. Therefore, we next used all the data to construct the neural network model.

**Table 1:** Five-time cross-validation results.

	**AUC**	**Accuracy**
FoldValidation 1	1	0.986301
FoldValidation 2	0.987256	0.946667
FoldValidation 3	0.99095	0.958904
FoldValidation 4	0.990676	0.958333
FoldValidation 5	0.996246	0.972603

From the output results of the neural network model (Supplementary File 4 and Figure 5B), it can be seen that the entire training was performed in 1423 steps. The termination condition was that the absolute partial derivative of the error function was <0.01 (reaching the threshold). The output results show that the weights of the model ranged from −4.67 to 4.53. The weight predictions were 4.527373 (*HMOX2*), −4.7670777 (*CSDC2*), 1.478590 (*ZMAT1*), 2.332519 (*SERPINA3*), −4.522891 (*FREM1*), and 1.940819 (*LCN6*).

### Evaluation of AUC

Using the three independent verification datasets of GSE116250, GSE42955, and GSE84796, after the maximum and minimum standardized data processing, the three scores were calculated and their classification efficiency was evaluated, and the AUC were compared. The three scores were as follows: 1) neuraHF, the scores obtained by summing the DEGs identified in this study multiplied by the weights obtained in the neural network; 2) CD8K [9], and 3) TP53 [10], which are reported characteristic genes associated with HF diseases in the literature.

**Figure 6:**
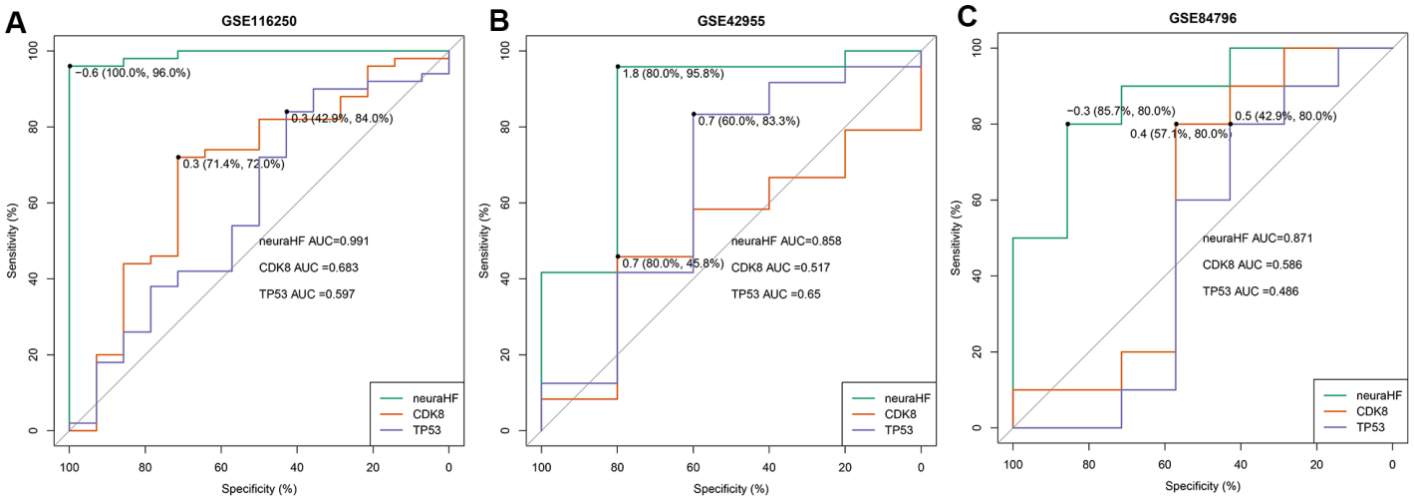
**Plot showing AUC verification results.** (**A**) AUC verification results in the GSE116250 dataset. (**B**) AUC verification results in the GSE42955 dataset. (**C**) AUC verification results in the GSE84796 dataset. The points marked on the ROC curve are the optimal threshold points, and the values in parentheses represent sensitivity and specificity. The AUC value is the area under the ROC curve.

Figure 6 shows a comparison of the three scores of the three independent verification datasets. In the GSE116250 dataset (Figure 6A), the AUC of neuraHF, CD8K, and TP53 was 0.991, 0.683, and 0.597, respectively. neuraHF had a sensitivity of 100% and a specificity of 96%. In the GSE42955 dataset (Figure 6B), the AUC of neuraHF, CD8K, and TP53 was 0.858, 0.517, and 0.65, respectively. neuraHF had a sensitivity of 80% and a specificity of 95.8%. In the verification results of GSE84796 (Figure 6C), the AUC of neuraHF, CD8K, and TP53 was 0.871, 0.586, and 0.486, respectively. The sensitivity and specificity of neuraHF were 85.7% and 80%, respectively.

## DISCUSSION

In this study, we calculated DEGs related to HF for the first time, and obtained six important candidate DEGs through the random forest classifier. We used a neural network model to determine the predicted weights of related genes, construct the classification model score neuraHF related to HF diseases, and evaluate the classification efficiency of the model score in three independent sample datasets. The AUC efficiency was excellent, and neuraHF was found to have a better classification efficiency compared with the other two HF-related biomarkers. However, because the weight predicted by RNA-seq used in constructing the neural network model was theoretically more suitable for disease classification of RNA-seq data, the GSE116250 dataset showed the best performance in the verification results. Meanwhile, because of the lack of the gene data for HFpEF in the GEO database, the genetic characteristics of HFpEF were not included in the construction of the diagnostic model, thereby compromising the diagnostic effectiveness of the model for HFpEF.

Of these six genes, *HMOX2* encodes heme oxygenase-2 (Hmox2), which is mainly expressed in the brain and testes [11–13]. Compared with heme oxygenase-1 (Hmox1), which has long been a focus of cardiovascular research, the study of Hmox2 is still in its infancy. It has been reported that Hmox2 plays an important role in oxygen sensing through the BKCa^2+^ channel in the carotid artery [14]. Meanwhile, Hmox2 also influences multiple biological processes by regulating the heme concentration in cells and the levels of CO and H_2_S. As an activator of soluble guanylyl cyclase, CO can activate the cGMP signaling pathway. In addition, the effect of CO on vascular relaxation also depends on the arrangement of the anatomical structure of the blood vessels and the relative ratios of heme oxygenase/CO and eNOS/NO. In addition, CO inhibits the production of the strong vasoconstrictor endothelin-1. Related studies have shown that CO can modulate cerebral blood flow by regulating the H_2_S pathway. However, most of these biological processes are achieved through first sensing of the O_2_ concentration by Hmox2 [15]. It is speculated that Hmox2 inhibits the systemic reactions in hypoxic diseases, but the specific mechanism remains unclear [15]. In general, oxidative stress is present in most cardiovascular diseases [16]. The specific mechanism is that a large number of cardiac cells (cardiomyocytes, endothelial cells, and neutrophils) can produce reactive oxygen species (ROS). Under normal physiological conditions, the heart exerts a defensive antioxidant function to maintain a dynamic balance with ROS generation. However, under the stimulation of pathological factors, this balance is quickly altered, and a large amount of ROS is released, causing peroxidation of functional proteins and lipids, and DNA damage, which leads to impaired myocardial contractile function and extracellular matrix remodeling [17]. Although Hmox2 is important to remove intracellular ROS, it plays an essential role in protecting cells from ROS-induced damage [18].

*SERPINA3* encodes serine protease inhibitor A3 (serpinA3), which is also known as α1 antichymotrypsin and is a member of the serpin superfamily. It plays an important role in the pathogenesis of various diseases [19, 20]. It activates immune cell functions mainly through influencing cathepsin G and elastase [21]. Interestingly, cathepsin G is present in large amounts in neutrophil granules and is mostly released during inflammation. However, long-term excessive release of cathepsin G can cause adverse reactions [22]. Inhibition of neutrophil accumulation in ischemic myocardium and continuous infusion of recombinant human α1 antichymotrypsin can significantly reduce the incidence of myocardial ischemia and reperfusion injury [23]. A proteomics analysis reveals that the serpinA3 level is elevated in the epicardial fat tissue of patients with HF and positively correlated with those of brain natriuretic peptide and C-reactive protein [22]. Furthermore, heart tissue of patients with HF can secrete a large amount of serpinA3 by itself, further increasing the intestinal tumor burden in mice [24]. These results implicate a role of serpinA3 in the development of HF.

The structurally conserved ligand-binding hydrophobic proteins of the lipocalin (LCN) family are widely represented in prokaryotes and eukaryotes [25]. Although LCN6 is highly enriched in human heart tissue [26] its function seems to maintain normal reproduction in male. However, there has been no relevant research exploring its role in the pathogenesis of HF.

More interestingly, during the analysis process of constructing a diagnostic model of HF, we identified for the first time that three key genes (*CSDC2*, *FREM1*, and *ZMAT1*) probably play a role in the pathogenesis of HF. Cold shock domain-containing C2 (*CSDC2*) is highly enriched in the human ovary, heart, adrenal gland, brain, and other tissues. The *CSDC2* protein encoded by this gene is an RNA-binding protein. Accumulating evidence shows that *CSDC2* is involved in the development of pyramidal neurons and maintaining normal decidualization in early pregnancy [27, 28]. FRAS1-related extracellular matrix 1 (*FREM1*) that encodes a basement membrane protein is highly expressed in the human endometrium and kidney. A. Mutation of *FREM1* causes nasal fissure with or without anorectal and kidney development abnormalities, suggesting a role in craniofacial and kidney development [29, 30]. Interestingly, after alternative splicing, the gene precursor encode and synthesize TILRR, an IL-1RI co-receptor that can enhance the recruitment of My88 and regulate Ras-dependent nuclear factor-κB amplification and immune inflammation [31]. Because of the significant activation of inflammation in the development of HF, this gene is likely to impact the pathogenesis of HF. Zinc finger matrin-type 1 (*ZMAT1*) is significantly enriched in human thyroid and ovary tissues and also expressed to some extent in heart tissues. There have been no reports on the function of this gene, but recent gastric cancer-related studies indicate that its long-chain non-coding RNA transcript variant 2 is associated with the poor prognosis of gastric cancer [32]. However, this study does not specify the biological function of the gene involved in the poor prognosis of gastric cancer.

The difficulty in obtaining heart specimens may reduce the potential application for HF. However, our present study does not intend to completely replace the existing diagnostic and treatment methods, but rather aim to supplement these methods. Generally, the current diagnostic criteria and procedures are based on data from patients with HFrEF. However, it remains unclear whether these are fully applicable to patients with HFpEF. For instance, it is difficult to diagnose mild symptoms of HFpEF using these noninvasive methods. However, the diagnostic model derived from our study can be applied to determine the possibility of heart failure by a timely cardiac biopsy. Therefore, our approach has a certain clinical value. Clearly, the accuracy of the model need to be investigated further in light of our present results.

## MATERIALS AND METHODS

### Data download and processing

The GEOquery [33] package was used for downloading data to obtain the expression profile and clinical phenotype data of chip datasets GSE57345, GSE42955, and GSE84796 and RNA-seq datasets GSE141910 and GSE116250, which are shown in Table 2. The respective annotation information of the chip probes of the corresponding platforms was obtained from the GEO database. During the conversion of chip probe ID and gene symbol, multiple probes were found to correspond to 1 gene symbol. In this case, the average probe expression was used as the gene expression level. The org.Hs.eg.db package (version 3.7.0) was used to perform gene ID conversion on the RNA-seq expression profile.

**Table 2:** Data download.

**Data**	**Sample size**	**Organization type**	**Data type**
GSE57345	313(Normal: 136; Disease: 177)	Non-Failing: 136	Microarray
		Heart left ventricle, idiopathic dilated CMP: 82	
		Heart left ventricle, ischemic: 95	
GSE141910	399(Normal: 166; Disease: 233)	Dilated cardiomyopathy (DCM): 166	RNA-Seq
		Hypertrophic cardiomyopathy (HCM): 28	
		Non-Failing:166	
		Peripartum cardiomyopathy (PPCM):6	
GSE42955	29(Normal: 5; Disease: 24)	Ischemic heart tissue: 12	Microarray
		Dilated heart tissue: 12	
		Normal heart tissue: 5	
GSE84796	17(Normal: 7; Disease: 10)	End-stage heart failure patients at the moment of heart transplantation: 10	Microarray
		Non-Failing: 7	
GSE116250	64(Normal: 14; Disease: 50)	Dilated cardiomyopathy: 37	RNA-Seq
		Ischemic cardiomyopathy: 13	
		Non-Failing: 14	

### Differential expression and enrichment analysis

The R software package limma [34] was used to conduct differential analysis on 136 normal and 177 HF samples of GSE57345. The limma software package uses the classic Bayesian data analysis to screen DEGs. The significance criteria for DEGs were set at a *P* value of less than 0.05 and logFoldChang (logFC) greater than 1.5. The pheatmap software package was used to draw the heat map of DEGs, and the R package clusterProfiler [35] was used to perform GO function enrichment analysis and KEGG enrichment analysis on related genes to identify three types of significantly enriched GO terms (*P* < 0.05) and significantly enriched pathways (*P* < 0.05).

### Random forest screening for important genes

The randomForest software package was used to construct a random forest model for the DEGs [36]. First, the average model miscalculation rate of all genes based on out-of-band data was calculated. The best variable number for the binary tree in the node was set as 6, and 2000 was chosen as the best number of trees contained in the random forest. Next, a random forest model was constructed and the dimensional importance value from the random forest model was obtained using the decreasing accuracy method (Gini coefficient method). The genes with an importance value greater than 2 and ranked in the top six were chosen as the disease specific genes for the subsequent model construction. The software package pheatmap was used to reclassify the unsupervised hierarchical clusters of the six important genes in the GSE57345 dataset and draw a heat map.

### Neural network to build disease classification model

Another dataset GSE141910 was selected for neural network model training. After the data was normalized to the maximum and minimum values, the R software package neuralnet (version 1.44.2) [37] was used to construct an artificial neural network model of the important variables. Four hidden layers were set as the model parameters to construct a classification model of HF diseases through the obtained gene weight information. In this model, the sum of the product of the weight scores multiplied by the expression levels of the important genes was used as the disease classification score. Caret (version 6.0) [38] was used to perform a five-fold cross-validation of the model results, the confusion matrix function was used to calculate the results of the five-fold cross-validation to obtain the model accuracy results, and pROC [39] software package was used to calculate the verification results of AUC classification performance.

### Additional data verification

The classification score model for the constructed HF diseases and the normal samples was tested for effectiveness verification on three independent datasets (GSE116250, GSE42955, and GSE84796). The pROC software package was used to draw three ROC curves for each dataset, and the area under the ROC curve was calculated to verify the classification efficiency. This was then compared with the classification efficacy of another two reported biomarkers of HF diseases. Meanwhile, the optimal threshold in the ROC curve and the sensitivity and specificity in classifying diseases and normal samples under this threshold were calculated.

## Supplementary Material

Supplementary Figure 1

Supplementary File 1

Supplementary File 2

Supplementary File 3

Supplementary File 4
