# Supplementary material for: Construction and analysis of a joint diagnosis model of random forest and artificial neural network for heart failure
Source: Aging (Albany NY). 2020 Dec 26;12(24):26221–35. doi: 10.18632/aging.202405 (PMC7803554; doi:10.18632/aging.202405)
Supplement: Supplementary Figure 1 [file aging-12-202405-s001.pdf]

SUPPLEMENTARY FIGURE

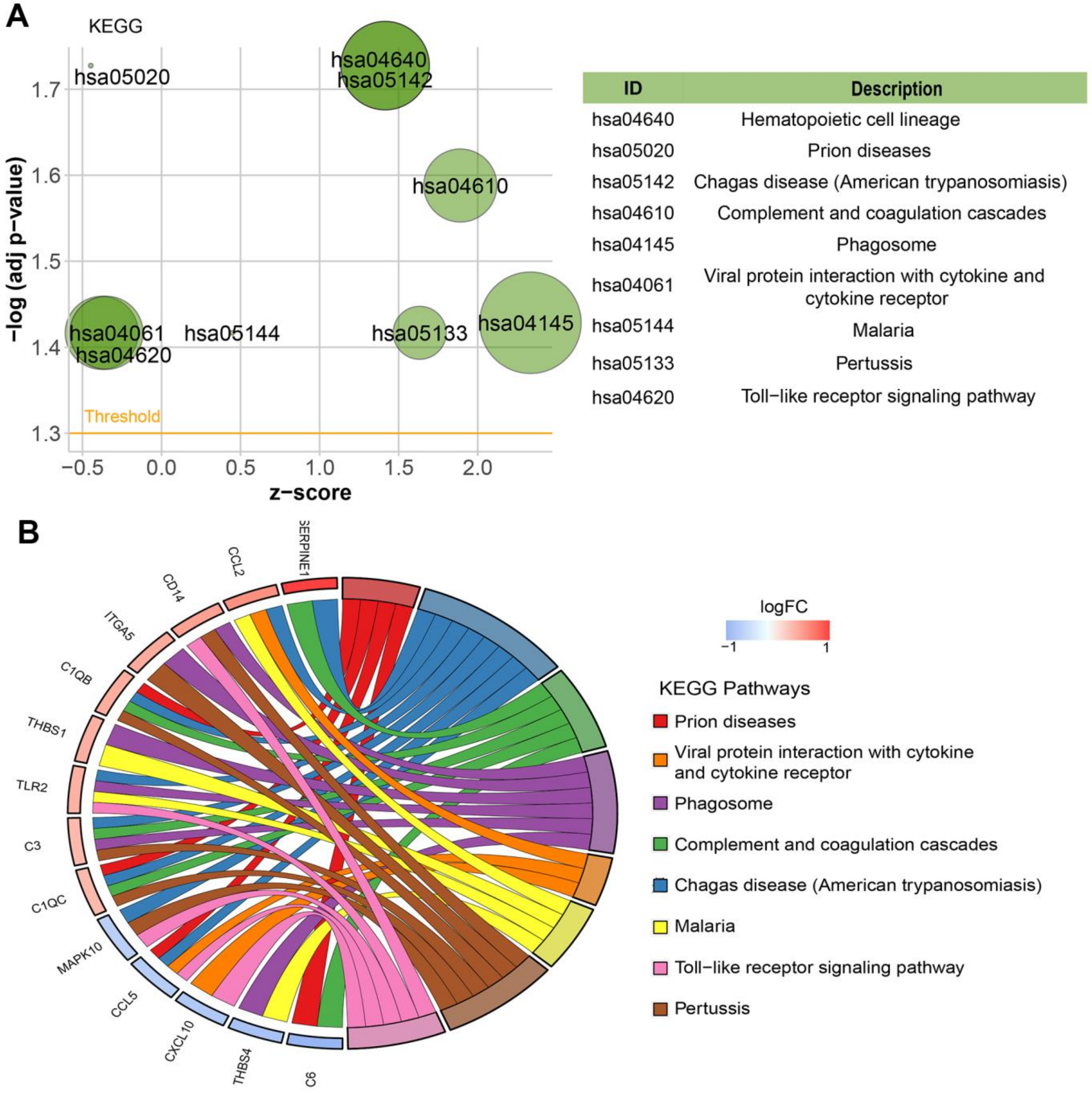

Supplementary Figure 1. Graph showing the KEGG enrichment analysis results. (**A**) Bubble chart showing the KEGG pathway enrichment results. The *x*-axis indicates the *z*-score, and the *y*-axis represents the −log10(adj *P*) value. A bubble represents a KEGG pathway, with the size of the bubble indicating the number of genes in the pathway. The pathway enrichment results of −log10(adj *P*) > 1.3 (*P*<0.05) in the figure are marked and shown in the table. (**B**) Ring plot showing the KEGG pathway enrichment. The left side shows the DEGs, the red gene band indicates upregulation, and blue indicates downregulation. The band on the right side with different colors represents different pathways. The connecting line indicates that the gene is involved in the pathway.
